# Web-Based Counseling for Problem Gambling: Exploring Motivations and Recommendations

**DOI:** 10.2196/jmir.2474

**Published:** 2013-05-24

**Authors:** Simone Rodda, Dan I Lubman, Nicki A Dowling, Anna Bough, Alun C Jackson

**Affiliations:** ^1^Turning Point Alcohol and Drug CentreFitzroyAustralia; ^2^Eastern Health Clinical SchoolMonash UniversityMelbourneAustralia; ^3^Problem Gambling Research and Treatment CentreUniversity of MelbourneVictoriaAustralia

**Keywords:** Internet, motivation, gambling, counseling, Web-based interventions, health services accessibility

## Abstract

**Background:**

For highly stigmatized disorders, such as problem gambling, Web-based counseling has the potential to address common barriers to treatment, including issues of shame and stigma. Despite the exponential growth in the uptake of immediate synchronous Web-based counseling (ie, provided without appointment), little is known about why people choose this service over other modes of treatment.

**Objective:**

The aim of the current study was to determine motivations for choosing and recommending Web-based counseling over telephone or face-to-face services.

**Methods:**

The study involved 233 Australian participants who had completed an online counseling session for problem gambling on the Gambling Help Online website between November 2010 and February 2012. Participants were all classified as problem gamblers, with a greater proportion of males (57.4%) and 60.4% younger than 40 years of age. Participants completed open-ended questions about their reasons for choosing online counseling over other modes (ie, face-to-face and telephone), as well as reasons for recommending the service to others.

**Results:**

A content analysis revealed 4 themes related to confidentiality/anonymity (reported by 27.0%), convenience/accessibility (50.9%), service system access (34.2%), and a preference for the therapeutic medium (26.6%). Few participants reported helpful professional support as a reason for accessing counseling online, but 43.2% of participants stated that this was a reason for recommending the service. Those older than 40 years were more likely than younger people in the sample to use Web-based counseling as an entry point into the service system (<italic>P</italic>=.045), whereas those engaged in nonstrategic gambling (eg, machine gambling) were more likely to access online counseling as an entry into the service system than those engaged in strategic gambling (ie, cards, sports; <italic>P</italic>=.01). Participants older than 40 years were more likely to recommend the service because of its potential for confidentiality and anonymity (<italic>P</italic>=.04), whereas those younger than 40 years were more likely to recommend the service due to it being helpful (<italic>P</italic>=.02).

**Conclusions:**

This study provides important information about why online counseling for gambling is attractive to people with problem gambling, thereby informing the development of targeted online programs, campaigns, and promotional material.

## Introduction

Internet interventions have the potential to cover large geographical areas at low cost and reach marginalized and difficult-to-reach populations [1]. Their potential for anonymity and convenience has increased access to information and counseling to groups such as young men [2], in addition to attracting new treatment seekers [3]. Particularly relevant to highly stigmatized disorders, such as problem gambling, Web-based (online) counseling has the potential to address common barriers to treatment, including shame and stigma [4,5]. Indeed, shame has been identified as a significant barrier to help seeking for problem gambling, as well as a reason for gamblers wanting to recover without formal assistance and not wanting others to know about the problem [6-8]. Research on Internet interventions and problem gambling has included investigating the effectiveness of self-directed Internet therapies [9], online peer-support groups and message boards [10,11], and tailored feedback on assessment [12]. With the exception of an evaluation of a UK program which provided the first publicly funded synchronous real-time chat intervention for problem gambling [13], no further research has been published on online counseling (ie, synchronous real-time chat) and problem gambling.

Research conducted in online counseling environments has typically attempted to identify similarities and differences to the therapeutic alliance found in face-to-face or telephone counseling [14]. Online counseling shares similarities with these other forms of counseling in that it is synchronous and involves at least 2 parties, but the lack of verbal, aural, and physical cues, argued to be critically important to the development of therapeutic alliance, is absent. Indeed, the disadvantages of online counseling have been well documented, and include a lack of audio and visual cues, limited capacity to develop a therapeutic alliance, and modality issues, such as typing speed, consent, privacy, and comfort with the medium [4,15,16]. Although legitimate concerns, it is possible that some clients find these issues attractive and as perceived benefits of online counseling.

The motivations of people involved in ongoing online treatment were captured in a small study by Cook and Doyle [17], who interviewed clients in their third or later session of predominately email counseling for a range of issues, including mental health and relationships. They found motivations for using email and chat included viability (believing this mode would be effective), disinhibition (lowered embarrassment or fear of judgment), cost, travel, the ease of developing an honest client-therapist relationship, and confidentiality/flexibility. In addition, clients said the benefits of a documented written text meant there was the capacity to read over and reflect on counseling sessions. This study has been used extensively as the basis for why people access online counseling, but the themes were based on the responses of only 9 participants, with just 3 engaged in real-time chat. Since this time, multiple studies have examined motivations of those engaged in appointment-based (often involving a cost) services and identified a number of additional motivators, including convenience, privacy and anonymity, face-to-face wait times, and access to specialized services [3,17-19]. To date, Leibert et al [18] conducted the only study to consider motivations as well as perceived advantages and disadvantages of using online counseling. This study found the reasons for using online counseling were similar to the perceived advantages (ie, anonymity, flexibility, emotional expression).

There is minimal research involving free online counseling without an appointment. However, investigations of motivations for using real-time chat provided without appointment have been conducted with clients and counselors of the Kids Helpline, an Australian telephone and online service providing counseling to young people. One of these studies attempted to identify the motivation for using online counseling over telephone or face-to-face counseling by recruiting young people waiting for a real-time chat counseling session into online focus groups [19]. A range of motivational factors emerged, including privacy and an emotionally safe environment (eg, reduced exposure, privacy, control), and issues around time (more time to reflect).

Although these studies are important in identifying why people use online counseling, there are several constraints that may limit their generalization to immediate interventions. These include relatively small sample sizes (some as low as 9 participants), as well as a lack of clarity on the modality of service offered (ie, typically email rather than chat). Studies have also tended to focus on the lack of face-to-face elements rather than possible benefits associated with their absence, and only 1 study involving adolescents directly asked about motivations for accessing counseling online over telephone or face-to-face services [19]. In addition, participants in these studies have predominately been drawn from ongoing appointment-based counseling, with limited research into the experiences of those accessing free services without appointment. Lastly, few studies have sought to explore motivations for using online counseling as well as the impact of their experience on reasons for recommending that modality to other people with a similar problem.

The aim of the current study was to determine reasons for choosing and recommending online counseling. Based on previous research examining ongoing clients involved in a range of online modalities (eg, chat, email), as well as young people engaged in brief interventions provided via real-time chat, we expected themes to emerge associated with anonymity, confidentiality, flexibility, and factors associated with the modality (ie, record of session). We did not expect that cost, therapeutic alliance, and counselor credentials, which are similar to telephone and face-to-face services, would be a reason for using or recommending online counseling over other options. In addition, recent research on barriers to help seeking for problem gambling [8] indicate shame and stigma to be barriers to engaging in professional and nonprofessional help among younger people (18-39 years of age). As such, we expected motivations for accessing online counseling would differ by age, with younger clients (<40 years) being more likely to endorse factors around shame and embarrassment than older clients.

## Method

### Participants

There were 241 participants with concerns about their own gambling, who accessed online counseling offered by the Australian national online counseling site Gambling Help Online [20] between November 2010 and February 2012. Six participants left both open-ended questions blank and were removed, leaving a final sample of 235 participants. Participants were more often male (57.4%) than female (42.6%), and ages ranged from younger than 30 years (30.6%), between 30 to 39 years (29.8%), between 40 to 49 years (20.9%), to older than 50 years (18.8%). Participants were most often engaged in nonstrategic forms of gambling, including electronic gaming machines, lotteries, bingo, and Keno (70.6%), than strategic forms of gambling, such as wagering, casino gambling, and sports betting (29.4%). A preference to gamble online was reported by 16.9% of participants. All participants were classified as problem gamblers as measured by the Problem Gambling Severity Index (PGSI) of the Canadian Problem Gambling Index (CPGI) [21] (mean 21.6, SD 4.0, range 8-27). Almost two-thirds of participants (62.1%) were new treatment seekers for problem gambling, with 33.6% having received counseling previously and 3.8% currently seeking treatment at another service. Participants with previous help seeking had accessed face-to-face (68.8%), telephone (15.1%), chat or email help from Gambling Help Online (9.7%) or other sources, such as international websites (6.5%). Most sessions occurred outside traditional business hours, including evenings and weekends (69.8%), and participants represented all states across Australia, except the Northern Territory.

Participants were offered an electronic exit survey at the completion of an online counseling session. The survey was provided as a link when a counseling session was terminated and was not promoted by the counselor or pop-up technology. The response rate for completing the exit survey was 17.1%. This response rate is comparable to online surveys that do not involve follow-up reminders, pop-ups, or other methods to increase participation [22,23].

To determine the representativeness of the current sample, the demographics of participants were compared with the total population of 1219 clients who completed a real-time chat counseling session with Gambling Help Online between November 2010 and February 2012. Chi-square (χ^2^) analysis indicated that there were fewer participants younger than 40 years (χ^2^_1_ = 27.1, *P*<.001), and significantly more participants who had previously sought counseling online, via telephone or face-to-face (χ^2^_2_ = 12.3, *P*=.002), in the research sample than the total client group. There were no significant differences between groups in terms of gender, ethnicity, severity, type, or mode of gambling.

### Procedure

Participants were offered an exit survey at completion of a counseling session via Gambling Help Online. This service provides real-time chat and email support to approximately 1500 people affected by problem gambling each year. Gamblers, family, and friends can access the service anonymously by completing a brief demographic survey and registering with an email address. Available 24/7, this service provides immediate free access to professional counselors without an appointment [24].

The service primarily provides counseling, information, and referrals for a range of gambling concerns. Brief interventions via reactive (inbound) helplines typically include brief assessment, feedback, and advice (eg, limiting time and money, scheduling alternative activities). Provided as single sessions, counselors responding to chat requests have qualifications in psychology or social work with training and expertise in the area of problem gambling. A typical session is delivered over a 45-minute period, although the amount of content covered online is approximately half of that discussed in face-to-face or telephone environments.

As part of a larger study, ethics approval was granted from the University of Melbourne’s Human Research Ethics Committee (ID: 1034028) and the Department of Justice’s Human Research Ethics Committee (JHREC) CF/10/17108. The exit survey was delivered at the end of the counseling session and contained 2 open-ended questions designed to elicit the motivations for choosing online counseling over other modalities. These included (1) What made you decide to use online counseling over other types of assistance (eg, telephone helpline, face-to-face counseling)? and (2) Would you recommend online counseling to someone concerned about a gambling issue (yes/no)? Why is that? The overall survey included a range of post-session indicators and took between 10 and 15 minutes to complete. On completion, participants saved the survey, which was stored in a secure online database.

### Data Analysis

The open-ended responses pertinent to this study were analyzed using content analysis [25]. This method was chosen because client responses were diverse (ie, 1 word to full-sentence responses) and we were interested in capturing novel and new themes as well as the extent of similarity of experience. In addition, this method of analysis allowed us to examine responses against previously developed categories and use an inductive approach to expand these new categories to represent the motivations of participants engaged in online counseling. Responses were capped at 200 characters and ranged between 1 and 52 words. Responses were typically brief, with participants responding with an average 11 words (median 8, IQR 4-15). When new categories emerged that were distinctly different to those previously reported, the researchers developed new labels and descriptions for these categories that were added to the dataset. Two of the researchers developed categories independently that captured all of the data (SR and ND), with a third researcher arbitrating differences and contributing toward the final category development (DL). To ensure categories were mutually exclusive, a number of categories were combined (eg, privacy and confidentiality) and subcategories developed.

Once initial categories were established, 2 researchers coded the entire sample and continuously checked categories with each other to ensure consistency (SR and AB). Responses varied from 1-word descriptions to sentences involving multiple reasons for using online counseling. As such, the unit of analysis was ideas or themes. For example, responses such as “access” and “I find it easier to access” were coded as *accessibility*. Responses including multiple ideas were coded into multiple categories. For example, the response “easy to use in comfort of home, safe, less confrontational” was coded into 2 categories, *access from home* and *comfortable*. Items that were ambiguous or not relevant to the motivation for treatment seeking were excluded from analysis (eg, “I have a gambling problem”). Participant quotes reported in this paper have been provided verbatim except with minor alterations to spelling. Words added to assist readability are indicated within parentheses.

Following the initial analysis to identify themes and develop categories, 2 raters undertook 3 hours of training in the application of the data dictionary (ie, definitions of categories and subcategories). They each coded 30 responses for the 2 open-ended questions (13% of the sample). As described by Neuendorf [25], the results of pilot testing were used to improve and adapt the coding dictionary before final coding and items with low responses were collapsed into single categories. Items were checked for interrater reliability using Cohen’s kappa (κ), which calculates percent agreement while correcting for chance. Scores of .41 to .60 indicate moderate agreement, .61 to .80 substantial agreement, and .81 to 1 almost perfect agreement [26]. A high interrater agreement was achieved ranging between .89 and .98. Eleven items were then resolved via consensus between the 2 raters, with a third researcher (DL) providing arbitration where consensus was unable to be reached.

To determine whether participants experienced online counseling differently according to age, gender, gambling type, and previous treatment experiences, data were analyzed via a series of chi-square procedures or *t* tests where data were continuous. A McNemar nonparametric test was used to determine change in reasons for use over reasons for recommending across the sample. Proportions reported throughout the results relate to the number of participants who freely reported each item rather than how many of the sample agreed with that reason.

## Results

### Participants

There were 222 participants who provided 351 reasons for using online counseling (13 participants did not respond to this question). Reasons reported by participants fell into 4 broad categories: (1) confidentiality and anonymity, (2) convenience and accessibility, (3) service system access, and (4) therapeutic medium. The same 4 broad themes emerged as to why people recommended online counseling, with the addition of access to helpful professional support. A total of 229 participants provided 311 reasons for recommending the service (6 participants were excluded due to insufficient information for classification).

### Confidential and Anonymous

Over one-quarter (27.0%) of participants mentioned issues around confidentiality and privacy as reasons for choosing online counseling over telephone or face-to-face counseling, and 21% stated that this was a reason for recommendation. For some participants, online counseling provided a discrete option that could be engaged in without others knowing. This may be due to the gambling itself being hidden from others or the act of seeking help being hidden. For some, online counseling provided a safe, private, and secure option where family, friends, or coworkers would not overhear the individual discussing the problem: “My phone bills are viewable by work or family; I don’t wish to be traced to calling for help” (male, 30-34 age group).

Anonymity was described as “not as daunting,” “not exposed,” and “not sharing problems with people you know.” It was viewed as an enabling factor to speaking about the problem, often for the first time: “It enabled me to face up to the fact that I have a gambling problem and talk to someone anonymously. It is the first time I have spoken to anyone about my despair over not being able to control my gambling” (female, 55-59 age group).

There was concern about being judged and embarrassment about having a gambling problem. Some participants described their experiences of having a gambling problem as a “disgrace,” and that they were “ashamed” and frequently embarrassed. For some, help seeking was “demeaning,” with 1 participant saying that they were able to admit the problem, but not accept the embarrassment of disclosure. For others, embarrassment had prevented exploration of phone or face-to-face options: “I feel very embarrassed about even ringing making an appointment and/or meeting someone face to face. After tonight I have more confidence about eventually consulting with a counselor; in the meantime, I feel it’s given me an avenue of help” (female, 60-64 age group).

Again, anonymity appeared to be an enabling factor for overcoming embarrassment and talking to a counselor online. Some participants separately related embarrassment to the benefits of anonymity and privacy. Indeed, when exploring why they would recommend the service, participants discussed feeling less judged and having increased control over the session: “It’s easy to be honest about feelings when anonymous. It is very difficult to talk about the extent that problem gambling affects all areas of life. Online appears less judgmental and the option is always there to switch off and run away if need be” (female, 55-59 age group).

### Convenience and Accessibility

Over half of participants (50.9%) stated that the reason they chose online counseling over telephone or face-to-face counseling was due to convenience, although only one-quarter (25.8%) cited convenience as a reason they would recommend online counseling to others.

Almost one-quarter (24.3%) of participants said they chose online counseling because it was easy, simple, flexible, convenient, and accessible. For some, easy access referred to being able to reach the service when experiencing difficulty: “The least effort to get counseling when you feel really down” (male, 30-34 age group).

Two time factors emerged, one around immediacy and the other around 24-hour access. Participants said they were attracted by the immediate and quick access to a counselor. In this situation, contact was typically in response to distress related to gambling behavior and wanting to speak with someone immediately. For others, accessing online counseling was a spontaneous decision that was facilitated by the absence of an appointment process: “It was available; I saw the literature at the club earlier tonight and thought I’d give it a go” (female, 40-44 age group, 3:14 am).

Similarly, 24-hour access was attractive as it provided a help-seeking option at a convenient time, including evenings, overnight, and weekends. A small number of participants described a preference for accessing online counseling from home. For these participants, the physical comfort and not having to go to an office was attractive. For others, online counseling provided a low cost option in which a landline was not available to call a helpline (mobile telephone calls to helplines are charged at standard call rate).

### Service System Access

Approximately one-third (34.2%) of participants stated the reason for using online counseling was related to service system access, although only 17.3% cited service system access as a reason for recommending online counseling to others. Specifically, 16.7% of participants identified online counseling as a good first step in both disclosing the gambling problem for the first time and accessing counseling: “I thought that it was a good place to start to get a feel for what I should and may expect from going to see a counselor face to face. It was a good first step and the online counselor provided information for me to go see a counselor” (male, 30-34 age group).

Sixteen participants cited dissatisfaction with other help (7.2%). This included a range of issues, such as wait lists or helplines not answering and unsatisfying interactions with counselors from other services. Some participants said they had tried everything else and were seeking a different perspective. In addition, 4 participants said that they did not know what other services were available or were not able to find any information on other options.

Referral to online counseling via advertising, word of mouth, and referral from other services was stated as a reason for using online counseling by 16 participants. Advertising and word of mouth were the reasons 11 participants came to the site, and included online and television advertising, as well as information found via search engines, gambling venues, or other websites. Two participants said that they chanced across the site and decided to “give it a go.” For 5 participants, online counseling provided a referral to other forms of help: “Initially did online counseling to enquire about face-to-face counseling. It was also an opportunity to experience it for the first time” (male, 35-39 age group).

Only 3 participants stated they used online counseling as an adjunct to other treatment. For these participants, it was a method of accessing support between counseling appointments, for relapse prevention, or when their counselor was unavailable.

### Therapeutic Medium

Thirty-three participants (26.6%) reported that they preferred online counseling to face-to-face or telephone counseling because of modality-specific features, with 17.9% citing these factors as a reason for recommendation. This included a preference to talk to, or through, a computer rather than face to face. For these participants, the experience of chatting online was viewed as easier than talking face to face or via the telephone: “It’s easier to talk to a screen” (female, 25-29 age group).

Participants identified a range of online counseling features as attractive, including the extended delivery time (ie, time to think and reflect), the capacity to review and save transcripts, as well as the act of writing over speaking. One participant also suggested this was a reason for recommending online counseling: “It’s less pressure. Writing actually makes you think about the situation in a logical [way]. Helps make order out of chaos” (male, 40-44 age group).

Eight participants reported that it was easier to express emotions using online counseling compared to telephone or face-to-face counseling. This was particularly the case where there was extreme distress and associated embarrassment: “It was late at night and I was very upset and crying so I would not have made any sense trying to talk to anyone” (female, 35-39 age group); “I do not like talking on phones and I prefer to cry without anyone seeing me” (female, 30-34 age group).

A few participants (4.5%) specifically stated that online counseling was more relaxed, comfortable, and less confronting than telephone or face-to-face counseling: “Less confronting at the moment, it’s easier when things are so bad to be anonymous” (female, 60-64 age group).

Lastly, 6 participants reported that the online platform facilitated more open and honest communication than phone or face-to-face modalities. This was also the case when participants reflected on why they would recommend online counseling: “It is discreet and allows for complete honesty with the anonymous counselor and yourself” (male, 30-34 age group).

In this case, the participant identified anonymity of the counselor as important. Indeed, a range of factors described previously, including anonymity, lack of physical presence, and the perception that they felt less judged appeared to facilitate honest communication.

### Helpful Professional Support

Few participants stated that they used online counseling because they thought it would provide access to professional and helpful support, but almost half of participants (43.2%) stated that this was a reason to recommend online counseling. Helpful professional support was highlighted as helpful for improving mood (eg, emotional regulation), confidence in resisting urges (eg, awareness of triggers), and addressing gambling cognitions (eg, alteration of gambling-related cognitions about winning): “I gained useful facts that opened my eyes and helped me realize that the machine is designed to make money and for you to lose it” (male, 20-24 age group).

Eighteen participants reported that they experienced the relationship with the online counselor as nonjudgmental and understanding, and indicated that the counselor knew what they were going through. Participants said counselors provided “thought-provoking questions” without “sugar-coating it.” Being able to access an independent/neutral professional was viewed as helpful in problem solving. In this situation, the counselor was viewed as empathic, expert, and credible: “Because I feel much better in myself and I didn’t feel judged in any way” (female, 30-34 age group).

Online counseling was recommended as a source of information and/or strategies. This included referral to other services and exploration of treatment options. Some participants described online counseling as “putting them in the right direction” and helping them to find the right resources: “They helped me out. They came back with answers; phone numbers, just general help. It was just nice to know there was someone on the other side, reading your problems and telling you their opinion” (female, 25-29 age group).

Overall, 87.7% of participants said that they would recommend online counseling to someone with a gambling problem. Fourteen participants (6.3%) stated that they did not like the medium or that it was generally unhelpful. Ten participants (4.5%) had specific issues with the counselor, involving miscommunication, a perceived lack of listening skills, or the delivery of empathy. Lastly, a small number of participants (2.1%) experienced problems with the technology itself, premature disconnection, or service dropout.

### Differences in Reported Motivations by Key Demographic and Help-Seeking Variables

We found few demographic differences between gender, age, gambling type (strategic and nonstrategic), preferred modality (face-to-face, online, or phone), severity of problem gambling, help-seeking experiences (new, current, or previous treatment seeking), time of contact, and reasons for using online counseling. However, those older than 40 years were more likely to use online counseling as an entry point into the service system (38.7%) compared with those under 40 years (26.1%; χ^2^_1_ = 4.2, *P*=.045), whereas those engaged in nonstrategic betting (eg, electronic gaming machines) were more likely to be motivated to use online counseling as an entry point into the service system (36.0%) compared with strategic gamblers (19.3%; χ^2^_1_ = 6.8, *P*=.01). Those older than 40 years were more likely to recommend it because of its privacy and potential for anonymity (25.8%) compared with those younger than 40 years (14.8%; χ^2^_1_ = 4.4, *P*=.04), whereas those younger than 40 years (47.2%) were more likely than those older than 40 years (31.2%) to recommend online counseling because it was helpful (χ^2^_1_ = 5.9, *P*=.02).

Given the similarities of response between motivations and recommendations, we were interested in the degree of movement between these 2 variables. In terms of movement between motivations for use and reasons for recommending, 84 (35.7%) participants changed their initial response about convenience when asked why they would recommend online counseling. Of these, 18 (7.7%) participants changed their response to convenience (from another motivation), whereas 66 (28.1%) did the reverse (χ^2^_1_ = 26.3, *P*<.001). For service access, 67 (28.5%) participants changed their initial response, with 49 (21.0%) participants subsequently not identifying it as a reason for recommendation, whereas 18 (7.7%) did the reverse (χ^2^_1_ = 13.4, *P*<.001). There was also a significant change in the proportion of participants endorsing helpful professional advice, with 93 (39.6%) participants subsequently endorsing helpfulness as a reason for recommendation, and only 3 the reverse (χ^2^_1_ = 82.5, *P*<.001). There was no significant change in the proportion of those endorsing anonymity/privacy or therapeutic medium. That is, those who endorsed these reasons did not significantly alter their response when asked why they would recommend online counseling.

## Discussion

### Principal Findings

This exploratory study provides a first look at the reasons why people choose online counseling over telephone or face-to-face counseling and why they would recommend it to someone else with a gambling problem. As expected, themes around anonymity and confidentiality emerged, as well as flexibility, albeit within the larger theme of convenience and accessibility. Openness of expression overlapped with a range of factors associated with the therapeutic modality, including a preference for writing instead of talking. Therapeutic alliance and counselor credentials, which are similar to telephone and face-to-face services, were not a reason for using online counseling, but access to helpful professional support as well as the development of a therapeutic alliance was a reason to recommend online counseling. The hypothesis that younger people would endorse more factors around shame and stigma than older people was not supported.

The findings of this study indicate that motivations for using online counseling over telephone or face-to-face counseling is in response to barriers, such as shame and stigma, and accessibility [7,27], but there are important differences, such as being able to easily reach help when experiencing distress or when highly motivated. Although our findings on age and gender by shame or stigma differed to those reported by Hing et al [8], we did find that males were more motivated than females by convenience and less by the immediacy the medium provided. In addition, younger people were also more likely to recommend online counseling due to its convenience than people older than 40 years. Clearly, the environment in which counseling is accessed is increasingly relevant in online counseling, where privacy is of concern during business hours, possibly when the individual is help seeking from a place of employment.

Congruent with previous research involving those engaged in a range of Internet interventions, concerns around anonymity and privacy, as well as easy and convenient access, emerged [10]. Anonymity in previous studies has been related to “perceived anonymity” [17], anonymity such that others are not aware of their treatment seeking [3], and being physically unseen by the counselor [18]. We found that anonymity was typically associated with an absence of identifying personal information (eg, their name), as well as both theirs and/or the counselor’s physical presence. Adolescents contacting the Australian Kids Helpline reported that the privacy of being online involved not wanting others to know that counseling was being sought at that moment (eg, late at night when others are asleep, or being overheard on the phone) [19]. These findings are also consistent with previous research, with online clients reporting greater concerns about their own physical environment (eg, being heard by someone else in the house) than helpline callers, who reported fears of the Internet being unsafe [28].

In addition to differences between face-to-face, telephone, and online environments, the current research identified important differences between immediate and appointment-based online interventions. Our study involved those accessing a free service that provided an immediate intervention. The intervention typically involves motivational interviewing and/or behavioral strategies [29] following a brief screen of gambling severity and the immediate impact of problem gambling (ie, level of distress). Although these therapeutic interventions have been found effective for problem gambling, there has been almost no research on the effectiveness of these interventions delivered at a time when the participant is eager, ready, and willing to talk. Indeed, participants talked about being highly motivated to act, which was often in response to distress, anger, or anxiety. A desire for emotional relief may partially account for the speed of immersion found in this and other studies [18,19], whereby the participant is already thinking about their concern prior to the counseling session commencing.

Despite participant uptake of anonymous online counseling, there is scant research on the clinical benefits of providing an immediate intervention. It is perhaps surprising given widespread funding for helpline and online services across most areas of mental health, that minimal research exists on the impact of providing an intervention at the moment the person is experiencing harm. Over the past 3 years, the advent of smartphones and other mobile devices has significantly increased the frequency of interventions occurring at the time of the event (eg, low mood) [30,31] rather than at some future time (ie, akin to appointment-based services). In addition, the utility of immediate interventions have also been explored in the context of emergency departments versus primary care, suggesting 85% of presentations relate to non-life-threatening issues [32]. In this setting, the presentation is not related to an accident or emergency, but patients present because of a range of factors, including convenience and access. A model by Padgett and Brodsky [33] of accident and emergency presentations suggests an interaction of predisposing issues (eg, social support), enabling factors (eg, accessibility), and perceived need (eg, level of distress), and partially explains why our participants would choose online over telephone or face-to-face services.

Few participants chose online counseling because it would provide helpful professional support, but when asked why they would recommend it to someone with a gambling concern, the fact that the intervention helped became important. Previous research involving clients in ongoing treatment indicated viability was a reason for choosing online counseling [17]. Although a few participants mentioned service viability as a motivator, the main theme that emerged in our study was related to helpfulness of professional support. Given that it could be expected that telephone and face-to-face services also provide helpful professional support, we suspect that the immediacy of the intervention, that is at the right time and right place, was important.

Participants were primarily asked the reasons for recommending online counseling to obtain additional information related to their motivations. Indeed, responses were similar across motivations and recommendations, except for the emergence of helpful professional support. Understanding the characteristics and experiences in relation to movement between these variables could have practical implications in terms of clinician training and evaluating service effectiveness. For example, shame and embarrassment is repeatedly reported as a concern for highly stigmatized conditions, but there is minimal literature describing how interventions and services best address this issue. In practical terms, it would be helpful to know whether it is clinically more important to address the reason for presenting to a service (ie, anonymity) or to provide an intervention that addresses the presenting issue (ie, gambling). Indeed, our study indicated that some participants did not shift perspective (ie, were motivated by anonymity and also provided this as a reason for recommendation), whereas others did shift (ie, motivated by anonymity and would recommend online counseling because it helped).

### Limitations

This research is the first to explore motivations of an adult population accessing immediate, online counseling for problem gambling. However, there are several limitations that need to be considered. First, participants in this sample were older than the population from which they were drawn and more often had sought help previously. Individual experiences combined with a slightly older demographic may mean that the motivations of some groups, including younger people, were underrepresented. In addition, we had expected issues around shame and stigma to be more frequently reported by younger than older participants. Our sample was slightly older than the total online counseling population, but still younger than other research involving adult gamblers (ie, 61% of our sample was younger than 40 years of age). It is possible that stigma is an issue for any age group or that issues such as access and convenience are more relevant for this population.

Second, surveys of help-seeking motivations are bound by the context and source of participants. As with the current study, they are typically cross-sectional, not capturing shifting motivations to change or motivations or readiness to seek treatment (eg, influence of gambling harms, social pressure, and time since last bet). Indeed, most previous surveys have involved clients of face-to-face or helpline counseling services, or identified perceived barriers of individuals not currently seeking help. Typically, these surveys have not been offered at the time the help was being sought and were retrospective reports. Although our study examined motivations at the time the decision was made, it is possible these reports were biased by their experiences of accessing the service.

Third, whether motivations are better identified via open-ended questioning or rating scales needs further investigation. As suggested by Pulford et al [7], many barriers to help seeking for gambling are not identified until prompted. Our study found approximately half of the sample were motivated by convenient and easy access, but it is possible that this was important to a larger proportion of the sample. In addition, the use of qualitative research methods allows us to be fairly confident that we are representing the views of participants, but there are issues with drawing conclusions related to the impact of motivations on recommendations. For example, we found significant movement between motivations and recommendations on convenience, service access, and helpfulness, but no significant change in anonymity/confidentiality and therapeutic medium. It is possible experiences, including the degree to which participant expectations are met, influence reasons for recommendation. Often client surveys include a question about whether the service would be recommended, typically as an indicator of satisfaction or rates of referral. However, responses may well be a better indicator of what happened in the intervention and whether they met participant expectations, rather than as an indicator of referral, which is possibly unrealistic when highly stigmatized conditions are involved. Indeed, service usage statistics for Gambling Help Online indicate that fewer than 10% of clients state their knowledge of the service was derived via a referral from a family member, friend, or professional.

### Conclusions

In summary, we found that reasons for choosing online counseling over telephone or face-to-face services include issues of confidentiality/anonymity, accessibility, service system access, a preference for features of the therapeutic medium, and professional support. Given the rapid expansion of service systems in response to the opportunities presented by technology, it is timely to identify the motivations for using services so that they can be better targeted, promoted, and configured. Most front-end gambling services, including telephone and online, are established at least in part to refer people to face-to-face services, but our research suggests referral to services that cannot be accessed at a convenient time or place, or where a referral is deemed unnecessary, requires further investigation. Ultimately, this would require the development of an evidence base, which demonstrates the uptake, usage, focus, and effectiveness of all clinical interventions on offer. This should also identify the dimensions of the counseling session that contributes toward perceived helpfulness, including the impact of counselor qualifications and counseling methods (including session focus, therapeutic techniques, and mechanics of online counseling) on client outcomes. Given many online clients indicate that online counseling was not just a first step, but the only step in changing behavior, there is an urgent need to develop and evaluate online single session interventions. To do this effectively, the reasons people are drawn to different services need to be further examined.

## Abbreviations

CPGI: Canadian Problem Gambling Index
PGSI: Problem Gambling Severity Index
